# Seroprevalence of Anti-Hepatitis A Antibody Among 1 - 15 Year Old Children in Kashan-Iran

**DOI:** 10.5812/hepatmon.10553

**Published:** 2013-05-27

**Authors:** Abbas Taghavi Ardakani, Babak Soltani, Mojtaba Sehat, Somayeh Namjoo, Mostafa Haji Rezaei

**Affiliations:** 1 Department of Pediatrics, Kashan University of Medical Sciences, Kashan, IR Iran; 2 Department of Community Medicine, Kashan University of Medical Sciences, Kashan, IR Iran; 3 Trauma Research Center, Kashan University of Medical Sciences, Kashan, IR Iran

**Keywords:** Epidemiology, Hepatitis A, Antibody

## Abstract

**Background:**

Worldwide, hepatitis A is a common infection during childhood especially in developing countries. It can cause severe complications in adults and patients with underlying diseases.

**Objectives:**

This study was performed to determine the seroprevalence of hepatitis A in 1 - 15 year-old children of Kashan.

**Patients and Methods:**

This cross-sectional study was performed on 666 one to fifteen year-old children from health-care centers in Kashan city during 2012. Total antibodies against hepatitis A were measured in sera by enzyme-linked immunosorbent assay (ELISA).

**Results:**

Totally, 3.9% of children were seropositive. Mean number of family members was 3.92 ± 0.89. There was no difference in seroprevalence of hepatitis A relative to sex, family size, mean age and age groups.

**Conclusions:**

In this city, a great proportion of children are susceptible to hepatitis A and it’s complications at an older age. This decrease in seropositivity may be caused by elevated hygien level. According to our results hepatitis A vaccination is recommended at early childhood such as that of other regions where low prevalence of hepatitis A infection is found.

## 1. Background

Hepatitis A has a worldwide distribution (1) but it is usually a mild and self-limited disease (2) with no chronicity (3). Hepatitis A in children is usually asymptomatic while in adolescents and adults the clinical presentations are more prevalent (4, 5). Hepatitis A is highly contagious. Transmission almost always requires direct contact between individuals via the fecal-oral route (6). A rare complication of hepatitis A is acute liver failure and those at risk for this complication are adolescents and adults, patients with underlying liver disorders or those who are immunocompromised (6). Mortality rate increases with age, especially when older than 40 years old (7). Numerous global data indicate that with the welfare of hygiene and quality of life there is a shift in the epidemiology of HAV (hepatitis A virus) to older people, predisposing them to the risk of serious HAV infection (8). Estimation of hepatitis A seroprevalence in a population is very important to determine strategies for infection control (9, 10). The primary methods for prevention of hepatitis A infection are improvement of hygienic conditions and immunoglobulin to provide short-term protection. Hepatitis A vaccine is recommended for children living in areas with high and intermediate endemicity and those who belong to communities at high risk for infections and epidemics (11-13). Hepatitis A vaccination has not been incorporated in the routine vaccination program in Iran. In order to prepare the vaccination protocol, at first the hepatitis A epidemiology must be surveyed in different parts of Iran. Kashan district, with a population of 500,000, is located in a tropical desert with an altitude of 982 meters from sea level. The temperature of this city reaches 43 degrees centigrade in summers (14). In this study we have tried to evaluate the seroepidemiology of hepatitis A in 1 - 15 year old children.

## 2. Objectives

The goal of this study was to estimate the seroprevalence and risk factors of hepatitis A in 1 - 15 year-old children in Kashan.

## 3. Patients and Methods

This cross-sectional and age-stratified study was carried out on 1 - 15 year old children in Kashan. The children were chosen from the only referral pediatric hospital in the area and five randomly selected public health centers (cluster random sampling) to reflect the portion of the population with lower accessibility to hospital services. If there were more than one family member with inclusion criteria, all of them were enrolled in the study. Study population was subdivided into three age groups, with 5 year range for each group. Weighted random selection of subjects in any age group was done according to the proportion of age groups based on the 2011 census. Children who had been recipients of blood or immunoglobulin during the last year, immunocompromised patients, rural and other nationalities were excluded. The questionnaires, which included age, sex and family size, were filled by a physician by interviewing the parents. Sample size was determined by estimation of 25% positive serology (15), confidence interval of 95%, power of 80% and design effect of 1.5. After written informed consents were obtained, 2 mL of blood sample was taken from each participant. Serum samples were stored at -20˚C until examination. Hepatitis A total antibody (IgM and IgG) was measured by a commercially available immunosorbent assay (ELISA) test. Quality control of kits was done at a specialized laboratory in Kashan. Specific antibodies present in the serum compete with specific antibodies linked to HRP (Horse Radish Peroxidase) enzyme to attach to antigens. By adding the enzyme substrate, the strength of the produced color will be reversely proportional to the quantity of specific anti-hepatitis A antibodies in the serum sample. The applied kit was produced by the American Trinity Biotech Company with sensitivity and specificity of 96%. Antibody levels more than the proposed cut off point specified by the manufacturer were considered as positive. Statistical analyses were performed by the SPSS version 17.0. The prevalence of anti-hepatitis A antibodies of each study group was reported. Findings were described by mean ± Standard deviation (SD) and proportion, and the distribution was presented by quartile and standard deviation. T-test, chi-squared test and Fisher’s exact test were used, considering a value of P < 0.05 as statistically significant.

## 4. Results

Totally 680 children were studied and 14 were excluded because they had received blood or immunoglobulin. From 666 children, two hundred eighty nine children were boys (43.4%) and 377 (56.6%) were girls. Two hundred seventy (40.5%) children were less than 5 years old, 215 (32.3%) were 5-10 years old and 181 (27.2%) were more than 10 years old and this represented the proportion of age groups in the total population as reported by the 2011 census in Kashan. The mean age was 6.98±4.02 years (Table 1). Twenty six (3.9%) cases were seropositive and 640 (96.1%) cases were seronegative and from the 26 seropositives, 12 (46.2%) were boys and 14 (53.8%) were girls (OR = 1.12 CI : 0.51 - 2.46). There was no significant relationship between seropositivity and sex (P-value = 0.772) (Table 2). 521 children had 1 - 4 family members and 145 had more than 4 family members. The mean family size was 3.92 ± 0.89. The mean family size in the seropositive group was 4.04 ± 1.07 and in the seronegative group was 3.91 ± 0.88 (OR = 1.55 CI : 0.52 to 4.58) . There was no significant difference in the mean family members between the two groups with a P value of 0.488. The relationship between family size and seropositivity was not significant (P value = 0.422) (Table 3). The mean age in the seropositive group was 6.02 ± 4.66 years and in the seronegative group was 7.02 ± 4.00 years. There was no significant difference in mean age between the two groups with a P value of 0.215; thus, no significant antibody positivity difference was detected according to age and age groups. The demographic characteristics of cases are indicated in Table 4.

**Table 1.** Frequency of Age Groups According to Sex

Age groups	Boys			Girls		
	Frequency	Percentage	Cumulative percentage	Frequency	Percentage	Cumulative percentage
**< 5**	126	43.6	43.6	144	38.2	38.2
**5** **-** **10**	97	33.6	77.2	118	31.3	69.5
**10 <**	66	22.8	100.0	115	30.5	100.0
**Total**	289	100.0	-	377	100.0	-

**Table 2.** Frequency of Hepatitis A Antibody According toSex

	Male, Frequency	Male, Percentage	Female, Frequency	Female, Percentage	P value
**Hepatitis A Antibody**					0.772^a^
**Negative**	277	95.8%	363	96.3%	
**Positive**	12	4.2%	14	3.7%	
**Total**	289	100%	377	100%	

^a^Pearson chi-square

**Table 3.** Frequency of Family Size According to Hepatitis A Antibody

	Seronegative		Seropositive		Total		P value
	Frequency	Percentage	Frequency	Percentage	Frequency	Percentage	
**Family size**							0.422^a^
**1 - 4**	499	78.0	22	84.6	521	78.22	
**4 <**	141	22	4	15.4	145	21.78	
**Total**	640	100	26	100	666	100	

^a^Pearson chi-square

**Table 4.** Demographic Characteristics

	Frequency	Percentage
**Gender**		
**Boy**	289	43.4
**Girl**	377	56.6
**Family size**		
**1 - 4**	521	78.22%
**4 <**	145	21.78%
**Age groups**		
**< 5**	270	40.5
**5 - 10**	215	32.3
**10 <**	181	27.2

## 5. Discussion

Hepatitis A virus (HAV) is a non-enveloped, RNA-containing virus that belongs to the family Picornaviridae. This spherical 27 nm diameter virus was discovered by Feinstone in 1973 (16). The infection is transmitted by fecal-oral and percutaneous routes. The incubation period is approximately twenty eight days. Fecal shedding rate of the virus is maximally during the late incubation period, several days before or shortly after the onset of symptoms (17). Following oral inoculation of a chimpanzee with HAV, the viral antigen was first detected in the serum on day 14, in the tonsils on day 16 and in the liver on day 21. The duration of viremia is about two weeks (18). In human surveys, HAV RNA is detected after an average of sixty days since the beginning of clinical symptoms (19). The risk factors that have been associated with transmission of HAV infection within the United States include sexual or household contact with another individual with hepatitis (25%), contact with a day-care center attendee (15%), international travel (5%) and food or water-borne outbreak (5%). However, in fifty percent of cases, no risk factor can be identified (20). The occurrence of hepatitis A seroprevalence is low to intermediate where 90% seroprevalence is only reached in adulthood (21). This indicates improvements in living standards over the past 10 - 15 years. Modalities based on hygien levels often fail to prevent spread of infection in outbreaks and vaccine is the best accessible tool (22). Due to a lack of sufficient studies in different parts of Iran, ascertainment of HAV epidemiologic properties are complicated and there is little information in this field. In our survey, the HAV seropositivity was investigated in 1 - 15 year old children, which was 3.9% in Kashan city. This finding may be exaggerated by our limited sample size, yet, this problem can be solved by increasing the sample size. Also some factors that may effect on serology of HAV were evaluated in this study. They included age, gender and family size, and none of them had a significant relationship with the seropositivity of HAV. In an investigation in Isfahan, 8.09% of children were HA seropositive and there was no association between family size, sex and positive antibody (23). In Zanjan the seroprevalence of HA was 44.3% in 7 - 10 year old children in 2007 and there was no serological difference between sex, age and family size (24). Our study was compatible with the two aforementioned studies in that there were no serologic difference between sex, age and family size. On the other hand, in a cross-sectional survey in the Fars province during March 2008 to March 2009 (in Shiraz and two other cities of Fars province) on 1050 individuals, IgG antibody against hepatitis A was positive in 88.2 percent of individuals. The seroprevalence in cases under 20 years was 79.3%, subjects 20-30 years of age was 91.3% and cases more than 30 years of age was 99 percent (P = 0.01). In this study, the seroprevalence was significantly associated with family size and rural residents (P = 0.001). Finally, they concluded that hepatitis A was highly endemic in their district and recommended hepatitis A vaccination at the same age as hepatitis B vaccination or at five years old (25). In European developed countries such as Italy, there is a substantially decreased in prevalence of hepatitis A infection, especially among the younger age group, due to marked improvements in socioeconomic situation and hygien levels. In this area, small outbreaks of HAV infection were associated with illicit intravenous drug abusers and those who had travelled to endemic areas, shellfish consumption and increasing number of family members (26). Contradictions in many investigations about the effect of risk factors on seroprevalence of HAV make interpretations difficult. In order to assess each factor, cases should be matched exactly to solve these controversies. So, further studies with a bigger sample size and appropriate matching of cases are necessary to determine the relationship of any risk factors with seropositivity of HAV. Most studies around the world, report that the HAV epidemiologic pattern is declining in seropositivity especially in the lower age groups. The statement about the change of epidemiologic pattern of HAV is feasible by comparing the seroepidemiology of HAV at two different time points in the same region. In this direction, during an investigation in Kuwait, the epidemiology of HAV (hepatitis A virus) in 1980 was in accordance with developing countries with approximately 100% seropositive adults aged above twenty years for hepatitis A. At the same time, 90% of the studied cases with acute hepatitis A were aged less than 10 years and 70% were below 5 years old (8). In another investigation in Kwait during 2003 to 2004, the seroprevalence of hepatitis A was 28% in adults and 25% of evaluated individuals less than 27 years of age were positive for antibody against HAV. This information showed that the change of epidemiology of hepatitis A in Kuwait has moved towards intermediate to low endemicity, getting 75% of the cases less than age of 27 years non-immune (27). No investigation on the seroepidemiology of HAV has been conducted in Kashan until now which makes it impossible to determine changes in serologic patterns of this district; thus, our study can be the first step for evaluation of HAV epidemiology in this city. It is noticeable that increase of socioeconomic and sanitary status leads to a decline of HAV infection and anti-hepatitis A antibody levels in the community, but the risk of HAV outbreaks will increase (28, 29). Studies in South-East Asia and China indicate that there is a shift in epidemiology of HAV from high to moderate or low endemicity. In China, there is a great risk of outbreaks as a result of transmission of the virus from the regions of high endemicity to low endemic areas with non-immune communities (30). In an investigation (2005) in Zabol (south-east of Iran), 100% of the 15 - 19 year old population were seropositive for HAV which categorizes this city as a hyperendemic area in our country (31) and preventative strategies seem to be mandatory for this region to control HAV infection and decrease the risk of outbreaks. Ximenes et al recommended hepatitis A vaccination for populations with low and intermediate endemicity in order to reduce the complications of hepatitis A in adulthood (32). Hepatitis A vaccine is highly purified and inactivated by formalin. It is shown to be safe and efficacious as implemented by Werzberger et al. (33) on seronegative 2 - 16 year old children in Monroe in 1992. Inactivated hepatitis A vaccine (VAQTA, Merck and Co Inc, West Point, PA, USA) is used in two doses (0 and 6-12 mo). The protection level of one or more doses of the vaccine is approximately 98%. Hepatitis A vaccine establishes long-term immunity, probably lasting 20 to 50 years (34). Since 1995, it has been accessible in the USA and is highly effective in preventing disease transmission in a population with repeated epidemics. The vaccine side effects are negligible and include fever, rash, redness and swelling in the injection site. The safety of this vaccine was proved with little adverse effects among 30,000 vaccine recipients (35). In a study, VAQTA hepatitis A vaccine was used in two doses for a group of infants at two years of age, who were followed up for 9 years and conferred long-term immunity. The vaccine was protective in preventing HAV epidemics in the population in spite of contact with sporadic cases in non-vaccinated individuals (33). Hepatitis A vaccination is not routine in Iran. Similar to our study, some investigations in different parts of Iran (23, 24) have indicated the low prevalence of hepatitis A in children, which is probably due to improvement of sanitation and socioeconomic status; Therefore, the necessity of hepatitis A vaccination in early childhood should be investigated and changes of immunization protocol against hepatitis A should be considered especially with regard to extensive travels between Iran and neighboring countries with high prevalence of hepatitis A, for prevention of epidemics. In this study, 3.9% of 1 - 15 year old children in Kashan city were seropositive. This shows a high rate of susceptibility in adults with regard to high prevalence of travels between Iran and neighboring countries with high endemicity of hepatitis A. Therefore, revision of national vaccination protocol is suggested and more comprehensive studies are mandatory to clarify the hepatitis A vaccination strategy. According to the low prevalence of hepatitis A infection in children of Kashan, seroepidemiologic survey of adults is recommended and if low prevalence is found catch up immunization in adolescents and adults may be prudent. Furthermore, in some cases, passive immunization (immunoglobulin) may be considered for prevention of hepatitis A. Finally, for a better evaluation of HAV infection prevalence and associated factors especially socioeconomic status, more comprehensive studies with larger sample sizes are recommended.
